# Book Reviews

**Published:** 1813-04

**Authors:** 


### CRITICAL ANALYSIS

### OF RECENT PUBLICATIONS

IN THE

### DIFFERENT BRANCHES OF PHYSIC, SURGERY, AND MEDICAL PHILOSOPHY.

---

*Practical Observations on Ectropium, or Eversion of the Eye-lids, with the Description of a new Operation for the Cure of that Disease; on the Modes of forming an Artificial Pupil; and the Description of some new Instruments and Operations for the Cure of Cataract, adapted to the different periods of life in which that Disease is found to occur. Illustrated by colored Plates. By* WILLIAM ADAMS, Member of the Royal College of Surgeons of London, Oculist Extraordinary to his Royal Highness the Prince Regent, Oculist in Ordinary to their Royal Highnesses the Dukes of Kent and Sussex, and late Surgeon to the West-of-England Infirmary for curing Diseases of the Eye, instituted at Exeter. 8vo. London, 1812. pp. 252. Callow.

THE work before us appears to be the result of a very extensive practical experience, from which source our author has drawn some highly important information, and made many valuable discoveries relative to those diseases of the eye on which he treats. With a laudable zeal for the improvement of that branch of surgery to which he now confines his practice, he has, in the present volume, communicated to the public this information, and these discoveries, in a perspicuous and candid manner, acknowledging, with becoming frankness, the difficulties he had to surmount in bringing the different operations to their present state of perfection, whereby those who may be inclined to practise the same branch of the profession, will be enabled fairly to appreciate the nature of the task they are about to undertake. Contrary to what too often happens in the present rage for book-making, this volume is composed of observations and inductions, drawn from the cases, which are fully detailed, and proportionally interesting and satisfactory; whereas it frequently happens that cases are formed to support particular hypotheses, and, although the leading particulars may be accurately stated, yet there is an evident bias to support opinions which the authors originally entertained. The first part of Mr. Adams's preface, given in his own own words, will, probably, better explain the nature of the work before us.

"In the following sheets, it has been a principal object briefly to describe my practice in the different diseases of which I have treated, and to relate the alterations and improvements in the operations, which experience has suggested to me. When I have spoken of the practice of others, it has been done solely to elucidate the subject in discussion, or to point out in what respect it either coincides with, or differs from, mine. I have, therefore, avoided swelling my book by numerous quotations, which tend rather to shew the researches of the author, than to increase the value of his practical remarks."

It would be well if this commendable feeling generally prevailed.

It appears that Mr. Adams, who is well known as the late surgeon of the West-of-England Infirmary for curing Diseases of the Eye, was the intimate friend, and confidential pupil, of the late Mr. Saunders. The death of this excellent practitioner was justly considered to be a great and serious loss to the profession; but it is a happy circumstance for suffering humanity that Mr. Adams having, for a considerable time, witnessed and assisted in all his operations both in public and private practice, and having also entertained an unreserved and friendly intercourse with him to the latest period of his life, has been thereby enabled to retain some facts of importance, not mentioned in Mr. Saunders's posthumous work, and even successfully to build a superstructure on some hints thrown out by him, which otherwise would have been lost to the world. We hope that Mr. Adams will investigate other diseases of the eye with the same industry and perspicuity he has evinced in regard to those of which he here treats, and that he will hereafter, as in the present instance, give to the public a candid and unostentatious account of the result of his experience.

From the originality and ingenuity displayed throughout this work, we do not think he has exhausted himself in the present publication, and should he adopt our suggestions, we shall not despair of seeing diseases of the eye considered in a less empyrical manner than heretofore, or of witnessing still more important improvements in their mode of treatment.

Our author has divided his work generally into three chapters; the first on ectropium, the second on contracted or obliterated pupil, and the third on cataract. These are again subdivided into sections, for the sake of order and perspicuity.

Chapter I.—The usual modes of operating for the cure of Ectropium, or eversion of the Eye-lids, as recommended by

Professor Scarpa, Mr. Ware, and the other writers on this disease, consisted in removing, with a curved pair of scissars, or with caustic, a portion of the conjunctiva on the inside of the eye-lid, in order to produce an adhesion and contraction of the remaining part, by which means the deformity and frequent inflammation occasioned by so great an eversion and exposure of the sensible conjunctiva, might be removed. These methods, our author informs us, he found ineffectual for a radical cure of the worst forms of the disease, as some degree of eversion still remained, which gave rise to repeated inflammations, the vessels becoming again turgid and overloaded, thereby re-producing the disease in as great a degree as before any operation. Mr. Adams then proceeds to state, in the following words, what led to the adoption of his present mode of practice.

"Mortified at having myself foiled, like others, in the removal of this deformity, I endeavoured, soon after the establishment of the West-of-England Eye Infirmary at Exeter, to which I was appointed surgeon, to devise some more effectual method of cure than any of those hitherto practised. With this view, I was led attentively to mark the appearance of the complaint in its most aggravated form, and to compare it with that of the eye-lids when in a healthy state.

"The edges of the tarsi, if the upper and lower lids are free from disease, when the eye is closed, are very nearly parallel; but when the eversion exists in a considerable degree in the lower lid, its edge is forced down towards the cheek, and forms a line of a crescent-like figure. In consequence of this morbid change in the parts, so much of the exquisitely sensible membrane of the eye-lid becomes everted, as to prove a continual source of pain and irritation to the patient, when exposed to heat or cold, sharp winds, dust, &c."

This comparison of the eye-lids, when in a sound and healthy state, must be admitted to be perfectly correct; but we have some doubts of Mr. Adams's mode of accounting for the production of the crescent-like curvature of the lower lid by an elongation of the tarsus, being equally just, as it appears to us much more probable, from the superior ductility of the integuments of the eye-lids to that of the tarsus, which is of a cartilaginous structure, that the integuments of the lids, and not the tarsus, are in fault. An artificial ectropium may be produced by drawing down the lid, which returns to its situation on the pressure being removed. We, therefore, would not say that the tarsus was elongated, because it is inelastic, but we should say that the integuments about the lid had been put upon the stretch. It is not surprising, however, that Mr. Adams should express himself as he has done, as his mode of cure consists in shortening the lid, by removing a considerable portion of the tarsus and lid in the form of a V, and afterwards uniting the two raw surfaces by the first intention, by means of a ligature. The part of the lid from whence this portion is to be taken, the different steps of the operation, and the after treatment, are fully described by our author in his work, to which we refer our readers.

The description of this operation concludes with an expression of surprise that, from its obvious simplicity, it should not have previously occurred to any of the eminent authors who have professedly written on the disease. Four cases are given in which it proved completely successful; and the colored engravings of the disease before the operation and after its removal, exhibit very satisfactorily the advantages obtained.

The preceding operation applies to simple ectropium, but the disease is sometimes found to exist in a more complicated form, when caused by cicatrices from small-pox, burns, scalds of the face, &c. Professor Scarpa was of opinion that a complete cure could never be expected in such cases, as he imagined that the portion of integuments destroyed could not be re-produced. Our author entertains a different opinion, and, although he does not appear to have performed the operation which he recommends for the removal of this species of the disease, yet, as the mode proposed is very ingenious, and likely, we think, to answer his expectations, we shall give it in his own words.

"In this species of the disease, I would recommend that a free separation of the integuments of the eye-lid and cheek should be made, and afterwards the lid should be shortened, and brought into contact in the same manner as in a case of simple eversion. By the speedy union of the divided edges, the eye-lid will be retained in its place, and the danger of the integuments adhering to the cheek, be in a great measure prevented. The extended raw surface would, most probably, be covered by a new production of skin; but should even some degree of shortening of the integuments occur during the progress of cicatrisation, and the edge of the tarsus be again somewhat pulled downwards, it will then be reduced to that stage of the disease where the caustic or scissars may be employed with advantage. Should any disposition to a relapse be observed during the progress of the cure, pressure may be made by means of a soft compress on the lid against the eye-ball, by means of a monocalous bandage; and the after treatment should, in every respect, be the same as that directed in cases of simple eversion."

The upper eye-lid is also subject to the same kinds of deformity as the lower lid, which prove even more distressing in their effects. Fortunately this appears to be a rare occurrence, currence, as in Mr. A.'s extensive field of observation for diseases of the eye, he has seen but one case of the kind; and he recommends the same modes of treatment to be observed as when the lower eye-lid is the seat of the disease.

Chapter II.—on contracted or obliterated Pupil,—commences with a concise history of Cheseldon's operation for artificial pupil; and quotations are given in it from many of the most celebrated authors who have written on diseases of the eye, which prove that the result of various trials of this operation both in England and on the continent, were so unfavorable, as to induce the most eminent surgeons and oculists to abandon it altogether as useless and inefficient. Mr. Adams has also given a detail of the circumstances which led to his adoption of this obsolete practice, proving, by unquestionable facts, that he communicated to Mr. Ware the favorable result of two operations of this kind eighteen months before the publication of the *third* edition of Mr. Ware's works on diseases of the eye, in which edition, although he speaks in high commendation of Cheseldon's operation (in direct contradiction to the opinion given in the *second* edition of the same work), yet Mr. A. complains, and we think with justice, that he has thus anticipated him in its communication to the public, without any acknowledgment that he was indebted to Mr. Adams for the first information of its successful revival. We regret this circumstance for the cause of science as well as liberality, since nothing can tend more to prevent that confidence which men of eminence in the same profession ought to feel towards one another, than the conduct just noticed. We, however, hope it will not influence that liberality of sentiment which has hitherto induced Mr. Adams to invite his professional brethren to witness his various and ingenious operations on diseases of the eye, whenever called upon to perform them; but, that, pursuing the same uniform line of conduct, his continued and well-deserved success will still further strengthen the favorable opinion entertained of his modes of practice by those who have seen the performance, and the result, of his different operations.

In the second are described different operations adapted to the various morbid states of the eye requiring an artificial pupil. These are for the simple obliteration of the pupil, obliterated pupil complicated with cataract, and obliterated pupil with opacity of the transparent cornea. The first is adapted to the formation of an artificial pupil after that aperture has become closed subsequent to the removal of the crystalline lens, which our author denominates a simple obliteration literation of the pupil. With a narrow one-edged knife, curved at its point like a dissecting scalpel, he makes a central division of the iris, two thirds the diameter of that membrane, by introducing the instrument through the coats of the eye, a line posterior to the iris, with the edge turned backwards, instead of downwards as recommended by Cheseldon; the point is then brought through the iris into the anterior chamber, and carried nearly to the nasal margin of the iris; it is then nearly withdrawn, making a gentle pressure against the iris with the curved part of its cutting edge, in the line of its transverse diameter. Instead of making considerable pressure with the instrument, and drawing it at once out of the eye, as recommended by Cheseldon, by which the iris was frequently separated from the ciliary ligament, Mr. Adams advises a very gentle pressure to be made use of, and to carry forward and withdraw the knife repeatedly until the opening is sufficiently extensive, when the radiated fibres contract, and form an opening of a large size.

When a contraction or obliteration of the pupil is complicated with an opacity of the crystalline lens, the primary steps of the operation are the same as in simple cases of that disease. The cataract is afterwards to be cut in pieces very freely, a part of which is recommended to be pushed into the anterior chamber, and the remainder suffered to remain in the newly made opening in the iris, which, acting as a plug, prevents the re-union of the edge of that membrane by the first intention. In the course of some weeks, when the portions of cataract are absorbed, all disposition to a contraction of the newly-formed pupil has subsided. When, from any accident, the lens is absorbed prior to the operation, but the capsule remains in a thickened and diseased state, if it can be divided in pieces, it is to be placed in the anterior chamber, that it may become dissolved: when that cannot be effected, it is to be depressed below the axis of vision.

In cases of cataract with closed pupil, Cheseldon supposed that the opake lens was of a smaller size than usual. Under this impression, he recommended the division of the iris to be made either in its upper or lower edge, by which means the light would not be obstructed in its passage to the retina. Mr. Adams very justly objects to this mode, first because the supposition that in such cases the crystalline was smaller, proves incorrect; and secondly, the rays of light being refracted towards the centre of the iris, during their passage through the cornea and the aqueous humor, vision must be proportionally indistinct according to the distance of the new pupil from that point. He adds, that in the majority of instances, the opake lens, so far from creating any difficulty in the execution or successful result of the operation, is actually serviceable, in as much as the fragments not only prevent a re-union of the divided iris by the first intention, but also assist them in their contraction, whereby an aperture of a sufficient size for the passage of light is obtained. This aid, as well as stimulating the iris with the point of the knife, is sometimes necessary, in order to excite the radiated fibres to act, when the disease has existed for any considerable period; and Mr. Adams remarks, in his general observations, that this contraction of the fibres sometimes occurs even two or three days after the operation has been performed.

The third subdivision includes the necessary steps for the formation of an artificial pupil, in case of opacity of the cornea, when the natural pupil is either obliterated in consequence of a protrusion of a large portion of the iris through an ulcerated opening in the cornea, or when the pupil is entirely obscured by a superficial ulceration of the cornea or capsule of the lens. In the first species of case, if the opacity does not obscure more than a third part of the cornea, a division of the iris should be made at the lower or outer part of the cornea, as in obliterated pupil complicated with cataract, a part of the divided lens brought into the anterior chamber, and the remainder used as a plug, by being interposed between the edges of the divided iris, in order to prevent its re-union. If the opacity occupies so large a portion of the centre of the cornea as to leave only one line clear at the margin, our author introduces the artificial pupil knife at the upper and outer part of the eye, and makes a vertical instead of a transverse division of the iris. This operation somewhat resembles that of Scarpa's, which, however, instead of dividing the fibres of the iris, detached the iris from the ciliary ligament next to the nose. When the pupil adheres but slightly to the cornea, in consequence of the iris being protruded in a small degree, Mr. Adams advises the artificial pupil knife to be introduced a little anterior to the iris, and its point carried on to the part where the adhesion of the iris to the cornea exists: the adhesion should then be divided, when the iris will recede to its proper place, the size of the pupil be again restored, and, provided the cicatrix in the cornea be not very large, the patient will recover useful vision. If, in addition to a small protrusion of the iris through the cornea, and its consequent adhesion to the cicatrix, there is a considerable contraction, or an entire obliteration, teration, of the pupil, the operation of a central division of the iris is to be performed, and the knife afterwards brought forward through the newly-formed aperture into the anterior chamber, and the adhesion of the iris liberated.

The extracts from our author have hitherto been confined to those species of the disease where ulceration through the whole substance of the cornea has taken place, which is always followed by a greater or a smaller protrusion of the iris. Opacities of the cornea often, however, produce a partial or total blindness, without any protrusion or adhesion of the iris. When the opacity is small, a drop of a strong solution of the extract of belladonna, applied daily, will afford very useful vision, by enlarging the pupil sufficiently to admit the rays of light by the circumference of the opacity. No apprehension, it appears, need be entertained from the long continued use of the belladonna, it being the opinion of our author, as well as Mr. Saunders, and the late Mr. Hill, of Barnstable (with whom, it appears, our author and Mr. S. served their apprenticeships), that its action is confined to the iris, and that it does not, in any degree, affect the retina. If the opacity of the cornea be so large as to prevent the dilatation of the pupil from benefiting vision, Mr. A. has recourse to an operation different from any he has yet described: his words are,

"I first fix the eye with the concave speculum, and then enter my closed pupil-knife through the cornea about a line anterior to the iris, and make the opening somewhat longer than the width of the instrument. Through this the aqueous humour will make its escape, and be followed by a part of the iris. If the iris does not protrude sufficiently, from the pressure of the speculum, to extend the edge of the natural pupil as far as the puncture in the cornea, I lay hold of it with a pair of small forceps, and gently pull it out, using great caution not to employ so much force as to rupture it. Having, in this manner, dragged the outer edge of the pupil a little through the puncture, I do not cut off the protruded part, but suffer it to remain strangulated, which prevents it from again returning within the cavity of the eye. The puncture heals, and it includes the protruded part of the iris, which is shortly removed by a very weak solution of argentum nitratum, dropped into the eye two or three times a-day. Care should be taken to make the incision no larger than just sufficient for the iris to protrude, in order to avoid the opacity which would be likely to ensue were it of a larger size, and also to prevent the iris receding when the cornea is again distended by the regeneration of the aqueous humor. In one instance, when there even existed a slight adhesion of the iris to the cornea, I was enabled successfully to accomplish this operation, after having first liberated the iris from its attachment."

The

The second Chapter, on artificial Pupil, concludes in the following manner:

"In the description of the different operations for artificial pupil, I have confined myself to the recital of facts ascertained by my own experience. It will be obvious that I have assumed the existence of circular and radiated fibres in the iris. This opinion, though supported by many eminent anatomists, is not generally considered as established. But it is foreign to the plan of the present work to discuss any theories upon the subject, for, whatever may be the result of anatomical observations upon this question, the practicability and success of the operations, as illustrated by the cases referred to, will stand upon the evidence of facts against all hypothetical reasoning."

The 3d and last Chapter is dedicated to Cataract, of which our author has taken a very comprehensive view, enumerating all the varieties of this morbid affection, and also describing instruments and particular modes of operating adapted to each of the different species of the disease. Mr. Adams says,

"In this, as in the two former chapters, I shall confine myself as much as possible to the recital of various facts and observations which have occurred to me in the course of my practice, and only add what is necessary for their elucidation."

It were a consummation devoutly to be wished, that all authors who profess to write practically would follow the same plan.

After describing the appearances of cataract in its different stages, Mr. Adams states it as his opinion that the disease is hereditary, unless where it proceeds from accident or from inflammation. In support of this assertion, he quotes the opinion of various authors, and adduces many very extraordinary facts which have occurred in his own practice. One of these was his operating on one of seven children, five of whom had congenital cataracts, and whose father and grandfather were both born blind of the same disease. Richter knew it happen in four successive generations, and these were not congenital cataract.

Syphilitic inflammation is reckoned one of the causes of cataract, in which species of the disease our author contends that the opacity is always at first confined to the capsule of the lens, though it may afterwards extend to the crystalline itself; and gives his opinion that it is only in this species of cataract that mercury has been found useful. He seems also to think that no external application possesses efficacy in curing the cataract in those cases where Mr. Ware has supposed ether to have been successfully applied. Mr. Adams remarks, that, as the opacity of the lens was in those cases produced produced by accident, it is more probable that the capsule, being ruptured (thereby causing the disease), it underwent a spontaneous cure by becoming dissolved in the aqueous humor and absorbed.

He mentions a case of this kind which occurred to one of the patients in the West-of-England Eye Infirmary, to whom, while waiting the gradual absorption of the cataract, he merely gave some calomel water as a collyrium, to convince the clergyman of the parish where the patient resided (and whose daughter, afflicted with solid congenital cataracts, had been advised by Mr. Ware to make use of ether to her eyes), that he was correct in attributing the cure which he predicted solely to the action of the aqueous humor. Mr. Adams quotes part of Mr. Cline's Lectures on Cataract, to prove the very great solvent powers which the aqueous humor possesses, dissolving the hardest substances when exposed to its action. The fact is so extraordinary as to be scarcely credible, were it not asserted on such undoubted authority, being no less than the dissolving of part of a knife, which, during the operation of extraction, broke off in consequence of a violent spasm of the muscles of the eye-ball, and stuck to the inside of the cornea. This important fact must remove, from the most sceptical mind, any doubts of its power in dissolving the hardest cataract when it is previously treated in the manner our author describes. Mr. Adams supports his opinion by the authority of Celsus, Bannister, Pott, Hey, and Scarpa, and mentions, as a conclusive authority, that Mr. Ware, since he saw him operate, has entirely changed the opinion given in his works on diseases of the eye, on the propriety of postponing any operation for cataract on children born blind until they should have attained an age to understand the necessity of keeping their eyes steady (the operation of extraction being impracticable at an early age); and, on the contrary, in a recent publication, strongly recommends Mr. Adams's operation for infants and young persons. In this publication, Mr. Ware certainly gives a decided preference to the operation of extraction, when the disease attacks adults or aged persons; but, as it appears he was then unacquainted either with the operation or the instrument which Mr. Adams recommends for the cure of cataract in old people, both of which are different from what he saw in the operation Mr. Adams liberally invited him to witness, Mr. Ware was certainly premature in giving so decided an opinion to the public, on the comparative merits of the two operations. Indeed, by a case adduced, he seems to have since successfully adopted for an adult the practice which he had previously condemned.

Although, by his recent change of opinion, Mr. Ware tacitly confesses the superiority of Mr. Adams's operation, still we should have been glad to have seen the acknowledgment from the pen of that eminent oculist, in return for the liberality with which that gentleman demonstrated before him a practice which, till then, Mr. Ware acknowledged he was entirely unacquainted with.

The second Section contains a description of instruments for the operation of the different species of cataract, and of the after-treatment to be observed. Our author commences with the following judicious remarks:

"Though a skilful operator may, doubtless, by his superior manual dexterity, supply many deficiencies in the form of his instruments, yet I conceive it to be incumbent on every surgeon, after he has determined on the mode of an operation, to consider attentively whether the form of the instrument generally used is well calculated to accomplish his object, or if it be capable of further improvement. This observation, though applicable to every operation which falls within the province of surgery, applies more especially to those intended to remedy the diseases of an organ of such extreme delicacy as the eye."

Mr. Adams comments on the different instruments recommended by Mr. Hey, Professor Scarpa, and Mr. Saunders, to which he objects as being ill calculated to accomplish his modes of performing the operations. He then describes his own two-edged needle for the cure of the solid cataract in children and adults. For the capsular cataract he prefers his curved pointed knife, which is much less bent than that of Professor Scarpa. The knife our author uses in the hard cataracts of old people, is the same as that he employs for the formation of the artificial pupil, only it is narrower. Mr. Adams describes the form of a new speculum for fixing the eye, which we agree with him in thinking much better calculated for the purpose than that of Pellier's, which is generally used. We will give his own description of this instrument, of which there are two views in neat engravings.

"The speculum of Pellier is, I believe, very generally used in this operation, but the patient often complains more of the pain caused by it than by the needle. This appeared to me to arise from the segments of two spherical bodies, nearly of the same magnitude, being in contact, by which all pressure is confined to a single point. Sufficient control over the action of the muscles of the eye-ball, can, therefore, only be obtained by using a degree of compression, which gives great pain during the operation, and sometimes produces a contusion of the coats of the eye, which I have seen excite and keep up considerable inflammation for some time. It is, therefore, evident Pellier's speculum culum is not well adapted for the intended purpose. The principle which ought to be kept in view in the construction of an instrument for fixing the eye, when the needle is used, is to divide the necessary pressure on as large a surface as possible, instead of confining it to a single point. To effect this, the bearing part of the speculum should be concave, and accurately adjusted to the convexity of the eye-ball, whereby the assistant or operator has much more command over it, and the ill consequences I have just mentioned are avoided. When a student at St. Thomas's Hospital, about six years since, I caused an instrument of the above description to be made of silver wire, similar to that employed in the construction of Pellier's speculum; but it failed to answer the intended purpose, as the curvature was irregular, and did not correspond with the convexity of the eye; its angles were besides so obtuse, as to prevent it from being easily inserted underneath the lid. Disappointed in my expectations, I have since had another made of solid metal, which possesses the advantage of a perfectly smooth and regular surface. The bearing part is so formed as to make an equal pressure on somewhat less than one third of the circular outline of the eye-ball; but, as this varies in different patients, the operator should always be provided with two or three instruments of different sizes."

All the species of cataract usually met with in practice are noticed under separate heads, and our author has, with great ingenuity, adapted operations to each, according to its greater or lesser solidity and complexity. The operations described are those for the solid cataract in children and adults, for fluid cataract, capsular cataract, adherent capsular cataract, solid cataract in old persons, adherent lenticular cataract, and capsular opacity with a transparent lens. As the modes of conducting the operations in these various species of the disease, as well as the instruments employed, differ materially from one another, it would be an injustice to our author to state any part without giving the whole, which our limits will not permit: we, therefore, refer our readers to the work itself.

An object of considerable importance has been accomplished by Mr. Adams, namely, that of ascertaining the characters and appearance of opacity in the posterior portion of the capsule of the crystalline lens, when this body itself, and likewise the anterior portion of the capsule, is transparent. He adduces two cases of this kind where he has effected a cure; one of these in a gentleman blind for eighteen years, and who had been considered by Mr. Ware to labor under amaurosis; the second, another gentleman blind for seven years from the same cause, and being a resident in London, had doubtless consulted the most eminent oculists;

 but but the real nature of the disease was not ascertained till Mr. Adams saw him.

The last division of this section is devoted to a description of the treatment proper to be pursued after the various operations.

Section 3d contains general observations on the different operations described, and shows, by the result of comparative trials, their superiority over all other modes of operating by the needle. The fundamental principle of our author's practice appears to be the bringing forward into the anterior chamber as many as is possible of the segments of the cataract, or even the whole of the lens *after its nucleus has been divided*, where its solution is found to be far more speedy than when suffered to remain *in situ*, and less inflammation succeeds the operation. By these means Mr. Adams generally succeeds at the first operation, and the inflammation in children is seldom so great as to require general depletion, and is easily removed in adults; whereas, when he performed the posterior operation of Mr. Saunders, he frequently found it necessary to repeat the operation four or five times, which was often succeeded by severe inflammation. To place in a strong point of view the effects produced by the different operations, our author claims particular attention to some cases detailed at full length, the heads of which he gives in the following words:

"It will be seen, by the case of Taylor (No. 36), aged 72, that, in the left eye, in which the nucleus was divided and placed in the anterior chamber, the whole of the cataract disappeared, without any bad symptom, in less than ten weeks; while in the right eye, where the lens was placed entire in the anterior chamber, it was nearly six months in dissolving, and great part of that time the patient suffered exceedingly from pain and inflammation.

"Mr. V.'s case (37), by two operations had the greater part of the anterior portion of the cataract sliced off, and placed in the anterior chamber, where it became absorbed in as short a period as is usual; yet, nine months after the first operation, it was necessary to extract the solid nucleus which remained undivided behind the iris.

"In the case of Coll. B. (38), although the cataract, denuded of its capsule, had been exposed to the action of the aqueous humor nearly five months, and subsequently remained in the anterior chamber for several weeks, yet the nucleus being undivided, it kept up a considerable degree of irritation, and in seven or eight months after the first operation, required to be extracted.

"In Mr. C.'s case (39), the cataract was of that favorable consistence which admitted of being at once very freely divided, and with so little pain that the operation was finished without his being sensible of its commencement; yet, as the fragments were not placed in the anterior chamber, they excited so much inflammation by their unequal pressure on the posterior part of the iris, that the destruction of the eye, or a closure of the pupil, was prevented only by the most active antiphlogistic plan. These unpleasant consequences are happily contrasted in the following cases:

"Mrs. H. aged 40 (case 42), in whom the cataract was of an unusually large size, and so hard that it could not be cut in pieces, but was merely divided in halves, and with the anterior part of the capsule placed in the anterior chamber, where they became absorbed in little more than five weeks. During this period, it was only necessary once to apply a few leeches to the eye-lids.

'In Anne Shee, aged 64 (case 40), the nucleus of each cataract remained behind the iris, deprived of its capsule and anterior part for ten weeks, without apparently diminishing in size, when, being freely divided by a second operation, and placed in the anterior chamber, each were in a fortnight almost completely dissolved, and the patient could then read the smallest print.

"The case of John Herbert, aged 40, (case 41) will be found the most striking and instructive. In his left eye, the cataract, which had existed for nine years, was divided into three or four parts, and the whole placed at once in the anterior chamber, where, without one unfavorable symptom, in five weeks it had entirely dissolved: in the other eye, one half of the cataract being also placed in the anterior chamber, had disappeared in about six weeks; while the other half, which remained behind the iris, was not completely absorbed in thirteen or fourteen weeks, during the latter part of which time it kept up almost constant irritation and inflammation in the eye, requiring repeated blisters, purgatives, &c."

Mr. Adams's observations on these facts appear to be so just, that we shall insert them instead of our own.

"The above cases clearly manifest that the quick solution of the lens, when thus divided and placed in the anterior chamber, prevents any considerable and continued inflammation: the necessity of repeated operations is also prevented. It is certain that a considerable degree of danger exists often, if the lens is suffered to remain behind the iris after it has been freely divided; and if it be not divided, whether it remains behind the iris, or in the anterior chamber, it will be indissoluble for a very considerable time, and not unfrequently require extraction. The inflammation resulting from an undivided lens, when placed in the anterior chamber, seems to arise more from long-continued friction than from the pressure of a large lens, as is particularly shewn in case 36, where the patient's sufferings seemed much greater when the cataract in the right eye was reduced to the size of a flax seed, than it was at first. In cases of the hardest cataract, and of the largest size, there is seldom any inflammation for the first three or four weeks after it has been placed undivided in the anterior chamber. During this period, its circumference and external surface dissolve as speedily as can be wished; but, the solid nucleus alone remaining, the irritation then commences: the ill effects may, however, be be avoided by the division or entire removal of the solid central part of the lens through a puncture in the cornea."

Mr. Adams's modes of practice are applicable to all ages, and appear to have been remarkably successful under congenital cataract, of which description of patients he has operated with the most favorable result upon more than seventy persons, laboring under double congenital cataracts, and has never failed but on two eyes, the pupils of which became obliterated in consequence of imprudent exposure to cold after the operation was perfectly executed. This is a success which would be accounted great in the most simple operation; it is therefore highly gratifying to find that one which has hitherto been considered the most difficult and uncertain in surgery, should have been brought to such a degree of perfection, and by means so little painful, as often to be performed even without the patient being sensible that it was begun. Our author, however, guards the operator against the mischiefs which may result from the attempt at the first operation to cut in pieces an opake lens in old persons, where its solidity forcibly resists the passage of the needle, as the action of the instrument may separate the capsule from the lower ciliaris, when the cataract will become partially depressed in the vitreous humor, turn round and press with its posterior convexity against the iris; or it will pass through the pupil into the anterior chamber. He then adds, "All these accidents occurred in my early practice, until experience enabled me to judge before the operation, from appearances, what might be the species of cataract, and its degree of hardness."

It would be well if all authors on surgical works were equally candid in acknowledging the difficulties unavoidably attendant on the perfecting a new practice, as it would point out to others in what manner they were to be guarded against and surmounted.

Mr. Adams strenuously recommends his modes of operation in preference to those of depression or extraction, as not only applicable to every species of cataract, which is not the case with either of those operations; but as being also exempt from the causes of failure which have contributed to bring them into disrepute, namely, the return of the lens to its situation, even after a lapse of years after depression, and the escape of the vitreous humor (even some days) after the operation of extraction, thereby causing the total loss of the eye, or an obliteration of the pupil. These accidents are not under the control of the surgeon, and have occurred in cases where the operations have been executed with the greatest prospect of success.

The

The unexampled success which our author experienced while surgeon of the West-of-England Eye Infirmary, seems most fully to bear him out in the preference he gives to his modes of operation; as he states, in a note, that, "during his residence at Exeter, he operated in succession on forty persons, of different ages, suffering under cataract, without experiencing a single failure."

Taking into account the contingencies to which the two operations of depression and extraction are liable, and which cannot be prevented by any degree of skill in the operator, we certainly are disposed to prefer the modes which Mr. Adams recommends, as they appear to us, while exempt from these dangers, to possess the advantages of both operations, namely, the facility of execution in the one, and in the other the certainty of a complete removal of the disease; indeed, in this respect, it is even more secure, as, from the capsule being cut up and absorbed, as well as the lens, not only is secondary cataract prevented, but also another cause of an obliteration of the pupil, both of which frequently succeed the operation of extraction, even in cases unattended with any discharge or lôss of the vitreous humor.

After pointing out the advantages of an early operation on patients afflicted with congenital cataracts, which our author proves may be successfully performed considerably under the age of twelve months, he gives some original observations on the different causes which retard their acquirement of the knowledge of visual objects after the cataracts have been removed, and points out some very ingenious means by which persons so circumstanced may be materially assisted in their progress of education. Mr. Adams's observations tend in a great degree to confirm those made by Cheselden, but he has inquired much farther than him into the causes of the curious phenomena observable in persons born blind of cataract, when cured. The Chapter concludes with a very deserving eulogium on the late Mr. Saunders, to whose instructions, as having been his friend and liberal preceptor, our author modestly attributes the information which led to the invention of the operations which are the subject of his work.

In a postscript are added some observations, in order to prove that the symptoms which hitherto have been usually considered diagnostic of amaurosis, in reality are not so, but frequently proceed from the retina having remained unexercised for a considerable time, in consequence of the existence of cataract. In proof of which Mr. Adams adduces several cases cases where his operation was successfully performed after the patients had been blind for a great number of years, and had been considered incurable by the most eminent oculists in the metropolis, who had pronounced them laboring under amaurosis.

---

*The New-England Journal of Medicine and Surgery, &c.* Vol. I. No. 3. Published Quarterly. Boston, July, 1812.

In continuing to notice this well-conducted Journal, we shall content ourselves with communicating from it such extracts as we think may interest our readers. The first paper contains an exposition of Sir Humphrey Davy's electro-chemical researches; the second treats of the spotted fever, as it has been inaptly termed. This disease has long occupied the serious attention of our transatlantic brethren; and, on a former occasion, we gave an account of it from the Massachusets Medical Communications.* In our climate there is little probability of this disease, if ever it should visit us, assuming a character so deadly as is the case in America; yet we feel interested in the examination which our medical friends in New England have instituted, to ascertain the nature of this malady; many of our readers, too, are not inhabitants of this country; and others of them, in the course of service, may be stationed in situations where a knowledge of this fever would be highly advantageous. From the essay before us, we find that the disease is not always accompanied with spots; for, in some of its epidemic attacks, this appearance has been observed in the proportion of one in ten cases. Neither is it, though termed a fever, always accompanied with pyrexiæ; but a few cases will best illustrate the peculiar nature of the complaint.

"*Case* 1.—A young mechanic of 19, firm in constitution and health, while playing at Morris, was *instantaneously* struck blind. *Immediately* followed nausea and sickness; next, a sharp pain through the temples and stomach; and, lastly, distraction: all in such rapid succession, that " in *five minutes*" from the date of the original blindness, the patient required four or five men to hold him. Three hours afterwards, (namely, at midnight,) his physician arrived, and found him still held by force to the floor; having excruciating pains in his head; and a pulse which was indeed feeble, but so frequent, as (in the phrase of the author) to " appear to be one continued stream." Within eight hours from midnight, the patient took 480 drops of laudanum, with a small quantity of diluted spirits, and obtained most essential temporary relief; his pulse being now " regular and in every respect good, excepting that it was yet too feeble." At night, how-

* Vide Med. and Phys. Journ. Vol. xxvi. p. 217—223.

ever, the patient relapsed into pain and into distraction, and was again restored by laudanum. We hear of nothing afterwards beyond debility, lowness, and occasional turns of light delirium, for a number of days; by which time, laudanum and diluted ardent spirits, with wine, had restored him to perfect health.

"Here was an attack without the cold fit of Dr. Cullen's definition. The mental agitations and bodily exertions of a man in a state of violent phrenzy, naturally gave immense *rapidity to his pulse;* and perhaps also produced *heat,* (though of this the reporter makes no mention.) The pulse, however, (for a time) recovered its natural *rate,* within eleven hours from the first attack; though it was still feeble, and continued feeble till convalescence occurred. In short, the seat of the attack appears to have been first in the head, with which the stomach instantly sympathized. We may also affirm, that the organs of sight suffered before either the stomach, the temples, or the intellect appeared affected."

"*Case* 2.—A delicate young lady of 15, rode 30 miles in a very cold day in a sleigh, [the sleigh* being probably without a cover.] Next day, 'she complained of having a bad cold;' and at her bedtime (which she fixed at a very early hour,) she was seized, *when going to her chamber,* with a severe pain in her little finger. When she had been a short time in bed, the pain *ascended along her arm and soon reached her head;* at which time she was taken with puking. Being subject to sick headachs, her servant encouraged this puking with diluted drinks. In the morning, the medical gentleman who had been sent for, 'found her in a very low depressed state, the whole surface [being] cold, and her pulse hardly perceptible.' The patient, after speaking only once during his visit, fell immediately into a profound sleep, which soon became the sleep of apoplexy; and, at the close of twenty-seven hours from the first attack, it was exchanged for the sleep of death: the affected hand and arm becoming black 'within five minutes from the time she breathed her last.'

"In the case of the preceding patient, we saw (as in this) the stomach sympathising closely with the head; but here the head being affected with stupor (instead of phrenzy), the whole surface was cold. It must not be said that the patient died *before* the cold fit could terminate; for, in the next case but one, the cold fit terminated in recovery, apparently without the aid of a *hot* fit. Again; in another of Dr. Strong's cases, as in the present, a symptom will be found operating like the *aura epileptica.* Lastly; in the case of another young female mentioned by Dr. Strong, a little motion in the cold air, by going from a sitting-room to a chamber, was instantly followed by an attack of the disease in its severest form."

* "English travellers speak of *sleds,* but more commonly of *sledges,* as carriages without wheels, employed in snowy countries for travelling over the snow. The citizens of the United States call such of these machines as are destined for accommodating travellers by the name of *sleighs,* and those used for heavy articles by the name of *sleds.*"

"*Case* 5.—A 'blooming' young lady (as she is called) of 11 years of age, who returned home from a neighbouring town [and probably in very cold weather], in order to be with her mother, then ill of the epidemic complaint; remained to appearance well, till the next day. Immediately after dinner, when going up to her mother's room, '*on the stairs,* she was taken with a severe *ague;* her teeth began to chatter, and her whole body appeared in a tremor.' She complained of an awful sensation of *faintness and coldness at the pit of the stomach,* and a peculiarly distressing *numbness* in one foot and leg. Her eyes looked *wild* and uncommonly brilliant; her *mind* was in a flighty state; her extremities cold; her pulse *feeble.* Brandy and laudanum were given, but wine being added, she threw up the whole, with her dinner in addition, nor could she from thenceforth retain any thing on her stomach for a moment. In two hours from her first attack, she became drowsy and comatose, and her throat was also *paralytic.* The disease resisting the only *remaining* means of applying remedies, her surface never became warm, nor did her pulse ever recover 'its energy.' After 15 hours from the first attack, (of which the last 13 were spent 'in a state of *profound coma,*') she expired;—just five hours after her mother, whole illness had lasted 52 hours."

From the result of all the cases adduced, it appears that in two only, shivering was observed in the beginning; heat occurred in none; and in four there was coldness or chill. The pulse was feeble, and in all commonly quicker than natural. In all there were nausea and sickness, and in two a sense of coldness and faintness at stomach. In all there was either pain in the head, mental derangement, or both. In all but one (who was much deranged) there was either coma or numbness, and in two a partial palsy. The evacuations were healthy. In one, carbuncles and pustules were seen; in another, apthæ. In all there was debility; and unless where there was phrenzy, the debility was usually from the commencement. From all these circumstances, and especially from the absence of heat, the author contends, that the disease does not come within Cullen's definition of fever. But rather than banish it from the class of fevers, he attempts a new definition of febrile disorders. Before he favors us, however, with this choice production, he clears the ground by getting rid of the most celebrated definitions of preceding writers, as Cullen, Linnæus, Vogel, Sagar, Fordyce, Darwin, &c. &c. proving that it is much easier to destroy than to create. The author's definition of fever, intending to comprehend the disease before us, is couched in the following terms: "An extensive morbid affection in the blood-vessels, *or else in their contents,* which sometimes discovers itself solely at intervals; and which commonly deranges one or more of the greater functions, as well of the body as of the mind, in a manifest manner." We do not profess to understand understand this assemblage of words, which the author means " should apply not only to cases of pure general fever, but to fever with a local type, and to symptomatic (that is, to accidental) fever." It appears to us, that the author is in the predicament of a wretched painter, who, having designed an animal, was ever obliged to write its name upon it, in legible characters, lest it might be taken for some other beast. No one could possibly imagine the preceding could be a definition of fever, had it not been introduced as such. It would apply as well to apoplexy; but it is faulty as a composition, and destitute of all the qualities of a definition, which especially demands to be clear and distinct, giving an accurate notion of the thing defined, whilst it should be so general as to embrace all the characteristics of the subject defined, and at the same time be applicable to none other. Having thus moulded his definition to admit the disease called spotted fever into the general order of fevers, the author endeavors to shew to what species of fever it belongs, and assigns to it the name of "*malignant nervous fever.*" He concludes the first part of the paper (for it is to be continued) by a recapitulation of the leading symptoms of the disease, which he remarks

" Sometimes occurs by a *sudden stroke*, as in apoplexy or palsy. Sometimes it is temporary apoplexy itself, in the shape of coma or stupor. Sometimes it is palsy in a paralytic limb, and sometimes it is the palsy of numbness; sometimes the throat is palsied; sometimes hemiplegia occurs; and sometimes it is on one side only that the vessels are affected. When sudden pains begin the disease, though they shift, they confine themselves in various instances only to one side. The head is generally the first part struck with pain; and the pain of other parts generally tends to the head, the head being rarely or ever free throughout from pain or some other affection, and, when the pain has been very violent, this affection is commonly delirium, and often phrenzy. Blindness, or some other peculiarity, and especially a dilated or contracted pupil, commonly attends the eyes; but the other senses, the feeling excepted, seldom suffer. The bodily powers seem dissolved. The chills are commonly general, with paleness; and, though *warmth* in the second stage of the complaint is spoken of, after warm remedies have been applied, yet nothing is said of general *heat*. The spirits are depressed, as in the deeper nervous affections. The heart seems distressed; and the pulse, after being first slow, soon quickens, but is always small and feeble, and sometimes highly variable. The stomach is irritable. There are frequent faintings. The breathing is laborious. Death may occur in 10 or 12 hours. Recovery from some of the worst cases has even in a few days sometimes only left behind a slight debility. The disease may attack more than once, as well as be attended with relapses.—Other symptoms will be noticed in another place: these regard the point at present under consideration."

 The

The next paper which claims our attention is by W. GAMAGE, Jun. M.D. *on the Effects of the Carbonate of Iron in the Ulcerated Uterus.* The preliminary observations are very judicious, and were suggested by Mr. Carmichael's Treatise on the Effects of Carbonate of Iron on Cancer. After commenting upon the impropriety of confounding diseases which have a resemblance only in some unessential particulars, the author observes,

"Very few of the cases reported by Mr. Carmichael, either in his Essay, or in the Medical and Physical Journal, and no one which exhibits his brilliant success, has, as we conceive, any well-marked relationship to the disease under which he chooses to rank them. They are, however, formidable in their character, and have not, we believe, been easily reduced to the control of medicine. We are, therefore, obliged to Mr. Carmichael for the increased ability which we gain over them, by the introduction of the preparations of iron as remedies; and he, probably, would have gained quite as much reputation, had he permitted each variety of disease to appear in its own natural garb.

"The object of this communication is not to criticise that gentleman, but to offer additional evidence in behalf of the carbonate of iron, not as a specific for cancer: experience commands the most decided protest against it, as a remedy in that disease, when, in consequence, any more effectual mean would be delayed. In two cases of cancer of the uterus, two of the breast, and in one of the parotid gland, it was administered with care and perseverance, without the least apparent benefit.* Opportunities of giving it a trial in all the variety of ulcers in which Mr. Carmichael used it, have not occurred to me; but I have prescribed it, with the happiest effects, in a dis-

* In one of the cases of the uterus, the disease was in a very advanced stage, previous to the administration of this medicine. In the other, it had progressed only so far as to evince its genuine character, but its advancement to a fatal termination appeared to be uninterrupted by the carbonate of iron. In this case, before the destruction of the patient, the scirrhosity had extended along the whole vagina, and, on one side, involved the inferior extremity of the external labium. In the one case of the breast, ulceration had taken place, and the disease had gone far beyond the reach of the knife: in the other, the glands of the breast were in the scirrhous state, the skin in contact with the scirrhous part, and a discoloration had commenced in it; but a probability of a cure by the knife still remained, could the patient have been persuaded to consent to the operation. In the case of the parotid gland, the disease had advanced too far for extirpation.

Besides the carbonate, I used in these cases the sulphate and the muriate of iron. I have not tried the phosphate nor the oxy-phosphate, which have been favorite preparations with Mr. Carmichael.

ease ease which I will take the liberty of denominating the phagedenic ulcer of the uterus.*

"This disease bears, in many points, a resemblance to the carcinomatous state of that organ, and especially in its advanced stages its effects are very similar. The most certain mark of distinction appears to me to be the different degrees of hardness produced in the part by each disease, the sensation to the touch arising from the hardness of the ulcerated, is so obviously different from that arising from the flinty hardness of the carcinomatous, state. Some of the most prominent symptoms of the ulcerated uterus, are soreness in the hypogastrium, bearing down pains, weakness in the small of the back, shooting pains from hip to hip, (this symptom is not always present, and when it is, it is not to be compared with the distressing lancinating pain which uniformly accompanies the carcinoma of this organ) some degree of enlargement of the os tincæ and fundus uteri, irregularity in the catamenia, and a constant leucorrhæal discharge. As the disease advances, great emaciation takes place; the countenance is sallow and distressed; pulse small, and feeble; there is a constant drizzling of blood, and a sanious discharge per vaginam; the labia of the os tincæ feel uneven; and the patient is subject to paroxysms of pains, like the parturient, accompanied with great hemorrhage."

Some cases illustrative of the disease are appended: our limits will not admit more than one as a specimen.

"*Case* 1.—In June, 1807, E. T. aged about twenty-two, applied to me for advice on account of severe pain and weakness in the small of her back, accompanied with a constant bearing down of the uterus. She had been afflicted in this way for a long time, and she said she had had, for a number of years, a whitish or yellowish discharge per vaginam, interrupted only by the catamenia, which had generally been irregular, in great quantities, and attended with indescribable sufferings. She was emaciated, countenance pale, pulse quick and feeble. Conceiving that these symptoms might be the effects of a long-continued leucorrhæa, I prescribed for that complaint. In the course of two or three months, she gained something in her general health, but there was very little amendment in her disease; the weakness, pain, and discharge, continued as at first. Suspecting a more serious difficulty than at first occurred to me, I made an examination per vaginam; this was unnaturally dilated, and there was some degree of prolapsus uteri; the os uteri was thickened, hard, and uneven to the touch; the fundus, as was ascertained by examination per rectum, was of a very unnatural size. I immediately prescribed the carbonate of iron in the form of an electuary, with conserve of roses in doses of half a drachm thrice in a day. In this dose it caused no inconvenience, and, after taking it for some time, she found herself

* One case, which appears to me to be of this description, is reported in the essay above mentioned, as successfully treated by the carbonate of iron. Mr. Carmichael, however, denominates it cancer of the uterus.

much much relieved. She continued to take it irregularly, however, for nearly two years; but I did not know the result of the case, till, in Nov. 1810, I learned, from her situation at that time, that she had no remains of her old complaint."

---

*A Treatise on Worms and other Animals which infest the Human Body; with the most speedy, safe, and pleasant, Means of Cure. By* T. Bradley, M.D. of the Royal College of Physicians, London; Senior Physician to the Westminster Hospital, and the Asylum for Female Orphans, &c. &c. 1813. pp. 213, and 3 Engravings.

It is well known that worms in the intestines are frequently the cause of very formidable complaints, both in children and adults: it is also well known that many persons are suspected to have worms in whom none exist, and the experienced practitioner has too often cause to lament that much positive injury has been done to the health, by the injudicious and violent means employed to cure a complaint which existed only in the imagination of the patient or his friends. This being the case, it must be obvious, that a treatise which should designate with accuracy the symptoms of worms, and point out appropriate remedies, would be a valuable present to the public; and this is what Dr. Bradley has here attempted.

To the work are prefixed some observations on cleanliness, the parent of health, to a want of which, and to improper diet, Dr. Bradley very justly ascribes the occurrence of worms, and of many other diseases.

It was the doctrine of Hippocrates, that worms were generated in the intestines of children while yet in the womb, and this doctrine has had many supporters among those who could not otherwise account for the manner in which worms first got into the body; but Dr. B. has never witnessed a single fact to countenance this opinion, nor does he think that the child is ever infested with worms so long as it is fed on the mother's milk *alone*. He considers worms as always arising from feeble digestion, aided by want of cleanliness, deficient exercise and improper food and clothing producing foulness in the bowels, and thus giving an opportunity to the eggs of worms, received into the stomach with the food, to be brought to maturity.

Dr. Bradley enumerates the following as the most constant and unerring symptoms of worms, viz.

"The belly being too hard, tense, and larger than natural; a peculiar disagreeable odor of the breath, especially of a morning before taking food; the tongue furred, notwithstanding the increased flow of saliva saliva into the mouth; a particular heaviness, or languid blueish appearance, about the eyes; a swelling and paleness of the lips, especially of the upper lips; itching of the nose, and sometimes a particular whiteness of it; pale, thin, crude urine, and in some instances of the color of whey, or quite white; the pulse sometimes hard, sometimes weak and quick, but always unequal; sour eructations; a sensation about the navel, which the patient, if old enough, endeavors to describe as a pinching or nipping pain; bowels very irregular, either obstinately costive or very loose; appetite also irregular, sometimes loathing all manner of food, at other times uncommonly voracious."

Dr. B. admits "that *other* diseases may produce the greater part, if not all, the foregoing symptoms, without the patient possessing a single worm of any sort whatever, and he instances, in particular, *hydrocephalus (apoplexia hydrocephalica)*, and *tabes mesenterica;* both of which, however, he thinks, may be distinguished from worms by the symptoms he has particularised.

Of the remedies that have been recommended as specifics for worms, there is an almost incalculable variety. From among these Dr. B. has selected such formulæ as his own experience has taught him to rely upon: the greater part of these are given in English, but such medicines as were thought more capable of doing mischief, if injudiciously administered, are prescribed in Latin.

Some observations follow on the *Guinea worm*, the *Clugger*, and some other insects infecting the external skin of the body; and, in an appendix, among other things, is given the anatomy of the intestinal worms, with references to the plates, which are neat uncolored copies from the beautiful delineations of these troublesome parasitical animals published by Dr. Hooper.

To persons not of the medical profession (for whose use this work is chiefly designed), and to young practitioners of medicine, this little volume may be safely recommended, as affording much useful information respecting a species of disease, which, though we believe it to be much less frequent than is commonly imagined, occurs often enough to claim a great share of our attention, and the treatment of which has been too often trusted to unprincipled pretenders, or to ignorant proprietors of active and sometimes dangerous anthelmintics.

---

*A Practical Treatise on the superior Efficacy and Safety of Dolichos Pruriens, or Cowhage, internally administered in Diseases occasioned by Worms; wherein are exhibited a concise Statement of the Symptoms of the Disease, and the Uncertainty*

*Uncertainty of most other Vermifuges now in use. To which are added, Observations on some other indigenous Anthelmintics of the West Indies; Testimonials of respectable Medical Characters on the Utility of Cowhage; and a Selection of Cases. By* W. CHAMBERLAINE, Member of the Royal College of Surgeons, London; &c. &c. The tenth Edition, corrected and enlarged. 8vo. Darton and Co. Highley, &c. 1812.

MR. CHAMBERLAINE's Treatise upon Worms is so universally known, that we have merely to state that in this new edition he continues to attest the powerful effects of Dolichos Pruriens as an anthelmintic. He is candid enough, however, to admit that in some cases of tœnia in which this remedy failed, Ol. Terebinthinæ, in the large doses lately recommended by some practitioners, has proved successful.

