# Corrigenda

**Published:** 1813-05

**Authors:** 


					314, ? 16, for " foiled," read " Tailed."
310, ? 6, for " C'heseidon," rend " Clielselden,"
317, ? 7, 13,37,Jor " Ctfeseldon," read " Cheselden."
321, ? 8, for " calomcl water," read "coloured water."
322, ? 27, for" curved pointed knife" read " curved pointea
needled
324, ? 43, read " Mrs. C 's case."
45, " Acr beiiij^ sensible."
320, ? 4, read 4i on Congenital Cataract."
22, for " iuwer ciliaris," rend " zona ciliaris."

				

